# Supplementary material for: The systematic position of the enigmatic thyreophoran dinosaur Paranthodon africanus, and the use of basal exemplifiers in phylogenetic analysis
Source: PeerJ. 2018 Mar 20;6:e4529. doi: 10.7717/peerj.4529 (PMC5865477; doi:10.7717/peerj.4529)
Supplement: Supplemental Information 1 — Includes results of all analyses, the character list for the updated Raven & Maidment (2017) analyses and the characters from Boyd (2015) that unite either Eurypoda, Eurypoda + Alcovasaurus or Stegosauria in Raven & Maidment (2017). [file peerj-06-4529-s001.docx]

Online Supplementary Material

CT-scanning methodology – page 2

Results of all analyses – page 3

Character list for the updated Raven and Maidment (2017) analyses – page 35

Characters from Boyd (2015) that unite either Eurypoda, Eurypoda + *Alcovasaurus* or Stegosauria in Raven and Maidment (2017) – page 38

CT-scanning methodology.

The previously referred teeth R4992 were CT-scanned at Imperial College London and a 3D model produced in SPIERS. The premaxilla-maxilla of R47338 was originally going to be CT-scanned as well, but the opening up of a fracture along the posterior process of the premaxilla meant it had to be treated in the conservation laboratory and could not be CT-scanned. The settings for the CT-scanning of the referred teeth are below:

Voltage - 100 kV

Current - 300 μA

Power - 30.0 W

Resolution - 5.102 μm

Results from all analyses.

Note that numbers below clades are symmetric resampling frequencies. Symmetric resampling was carried out with a probability of 33 and using 1000 replicates on a ‘New Technology’ search of existing trees, subsequent to the analysis being carried out.

Strict consensus tree of 3030 MPTs of Analysis A1


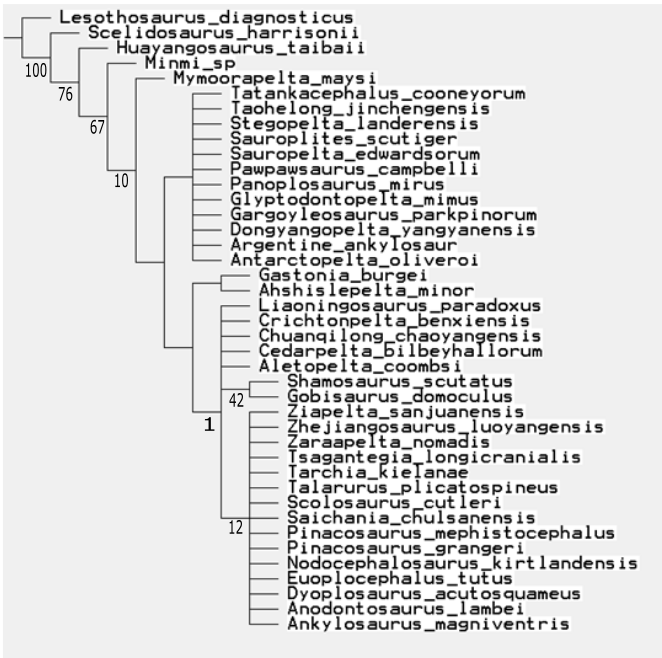


Strict consensus tree of 11 MPTs of Analysis A2


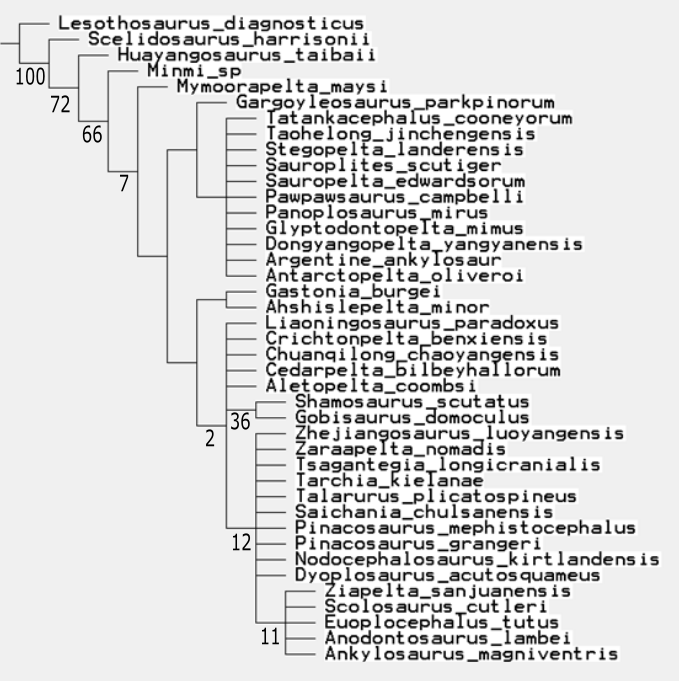


Strict consensus tree of eight MPTs of Analysis A3


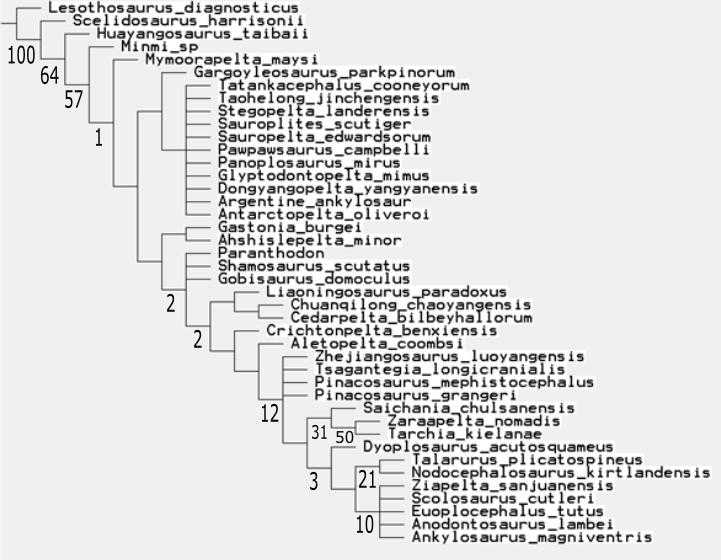


Strict consensus tree of eight MPTs of Analysis A4


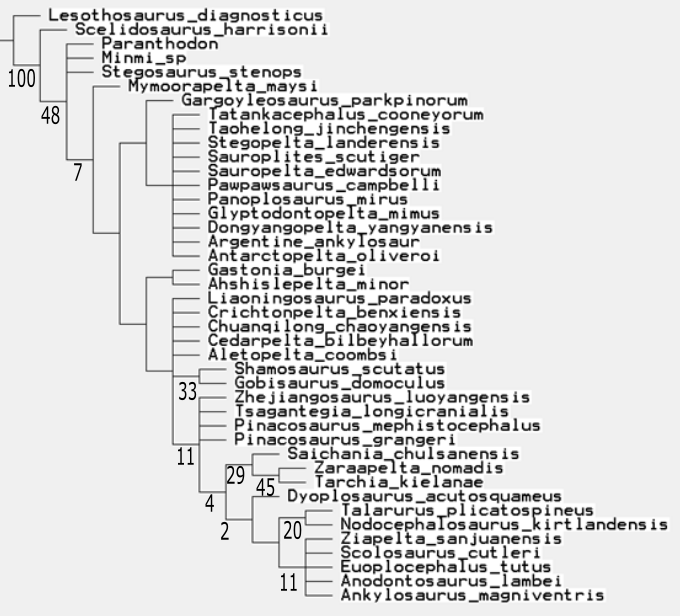


Strict consensus tree of nine MPTs of Analysis A5


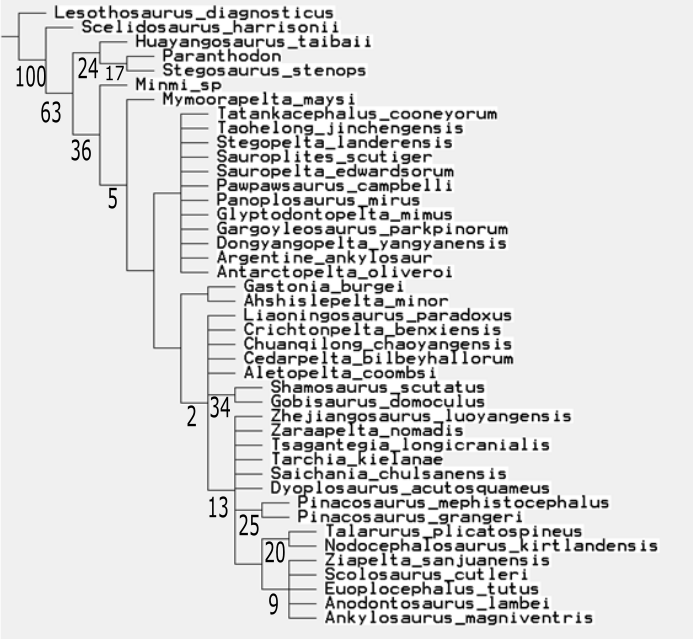


Strict consensus tree of nine MPTs of Analysis A6


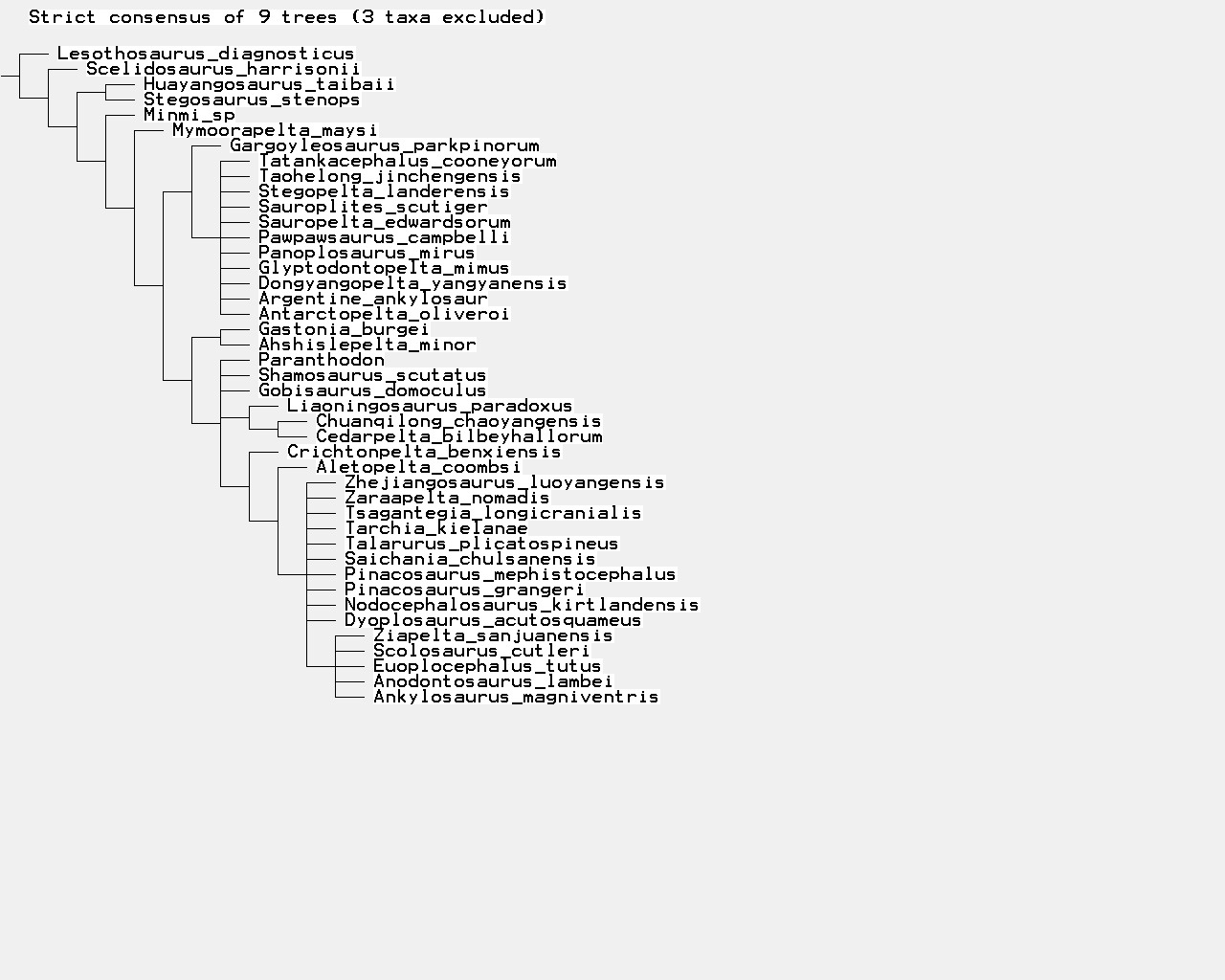


Strict consensus of 10 MPTs of Analysis B1


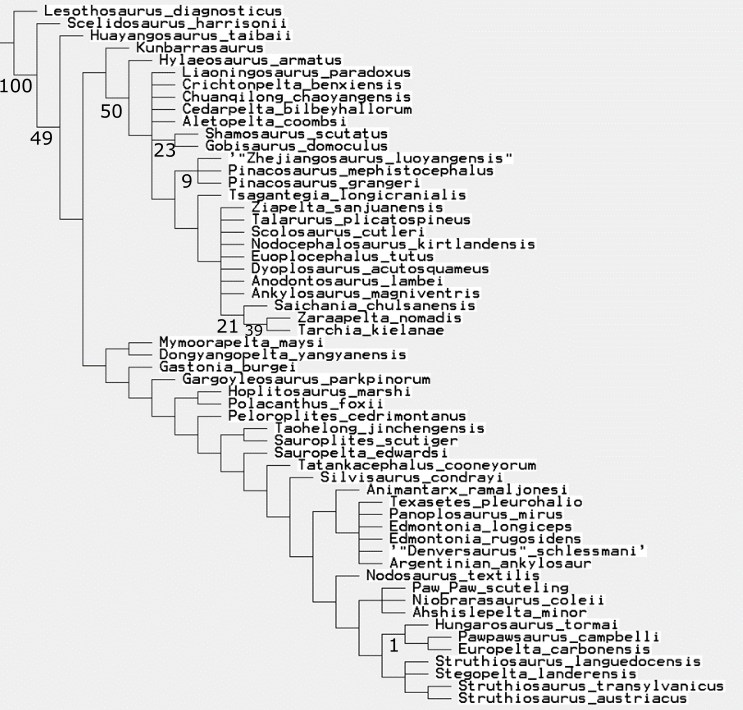


Strict consensus of three MPTs of Analysis B2


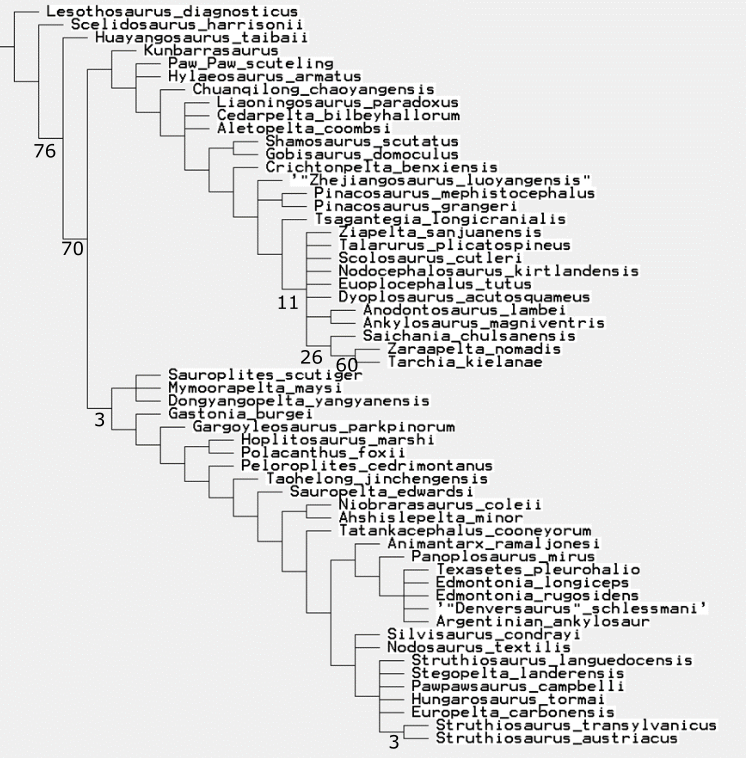


Strict consensus of three MPTs of Analysis B3


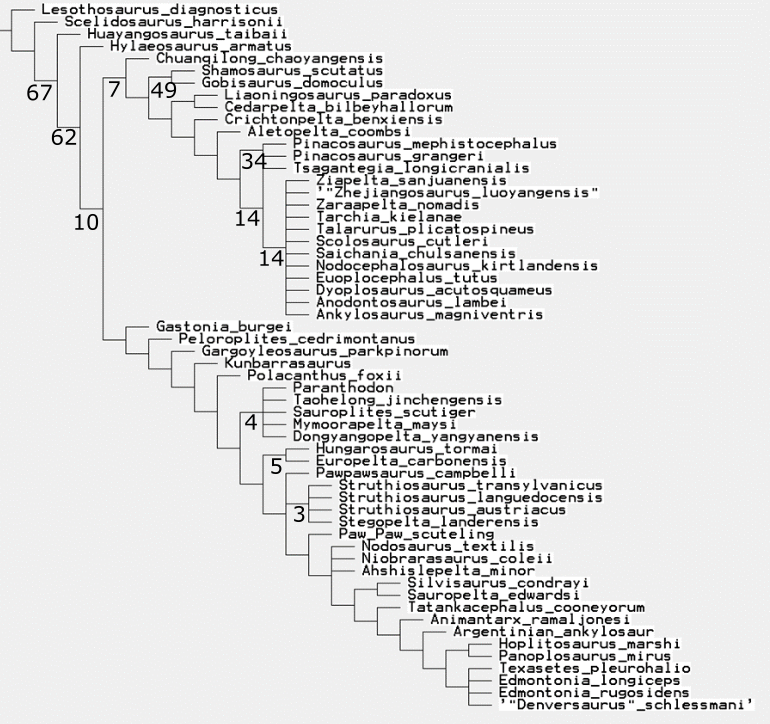


Strict consensus of five MPTs of Analysis B4


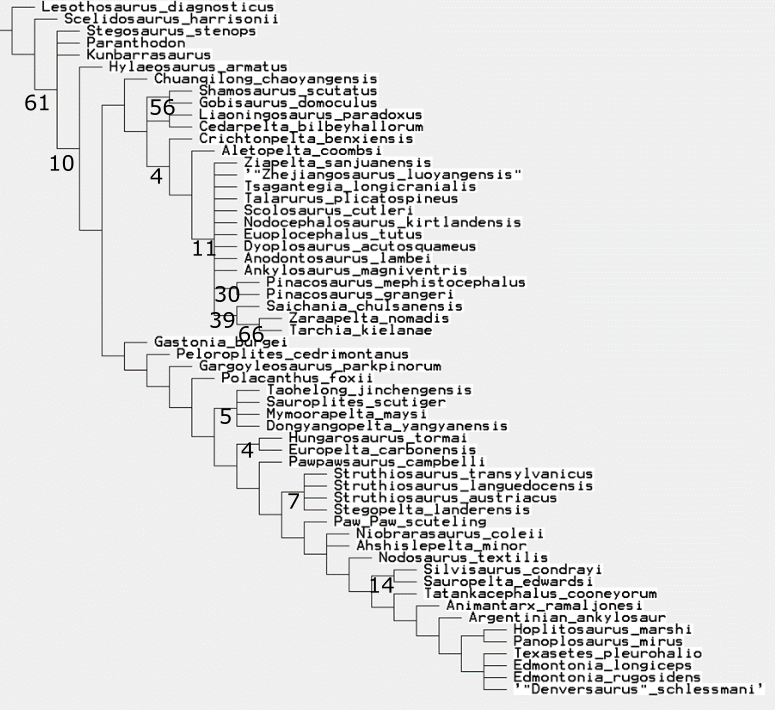


Strict consensus of two MPTs of Analysis B5


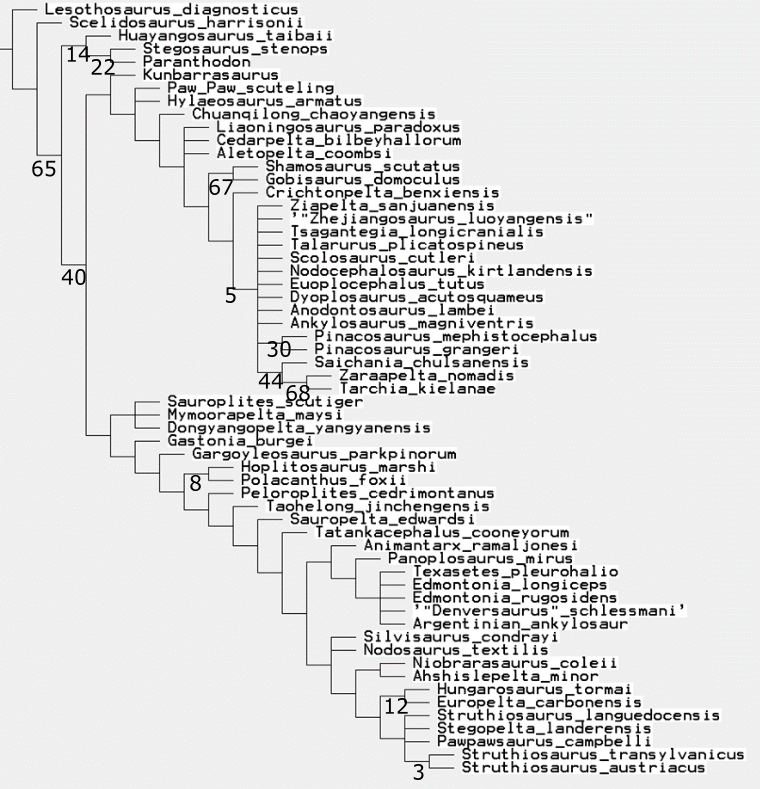


Strict consensus of three MPTs of Analysis B6


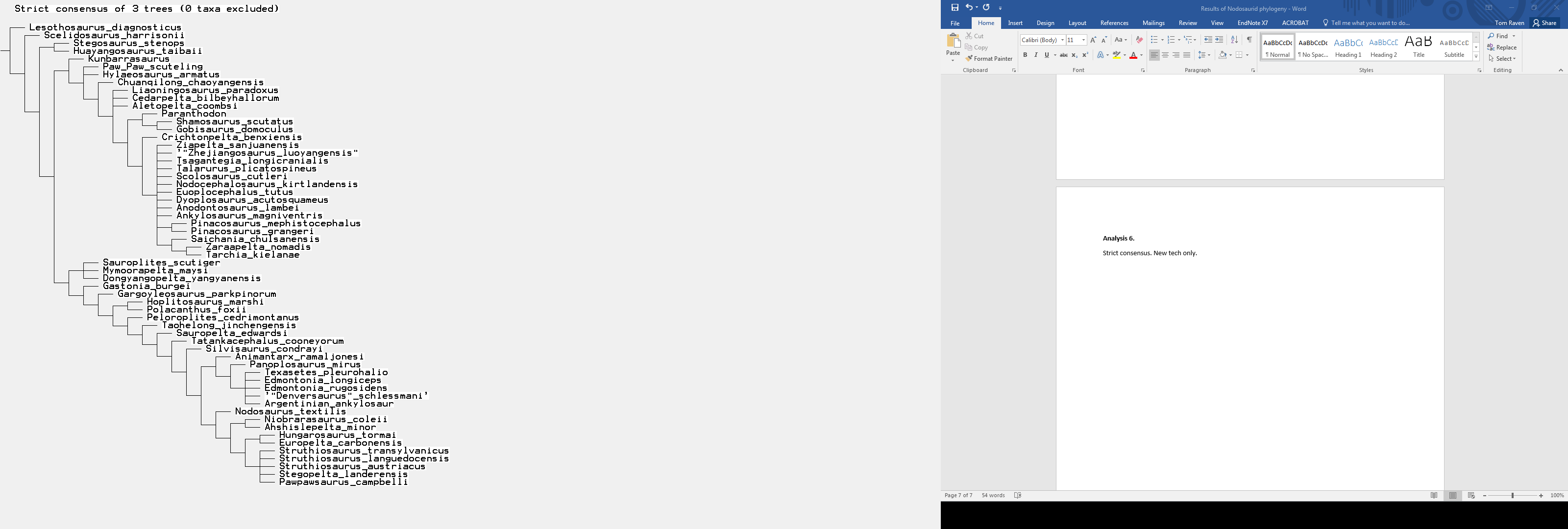


Strict consensus tree of Analysis C1


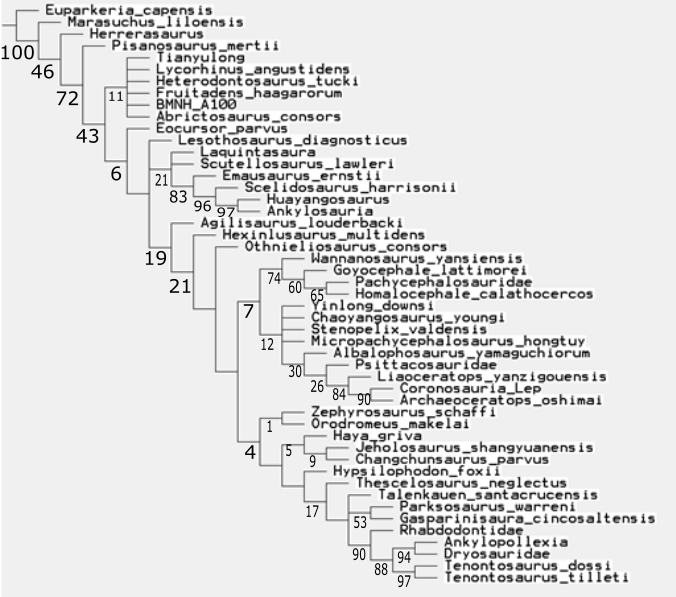


50% Majority Rule tree of 144 MPTs of Analysis C2


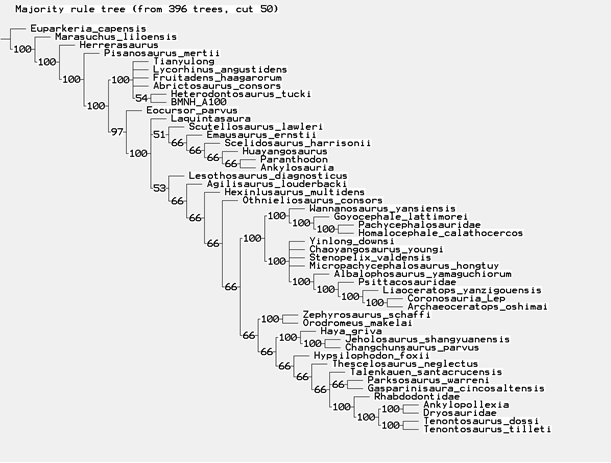


Strict consensus tree of 96 MPTs of Analysis C3


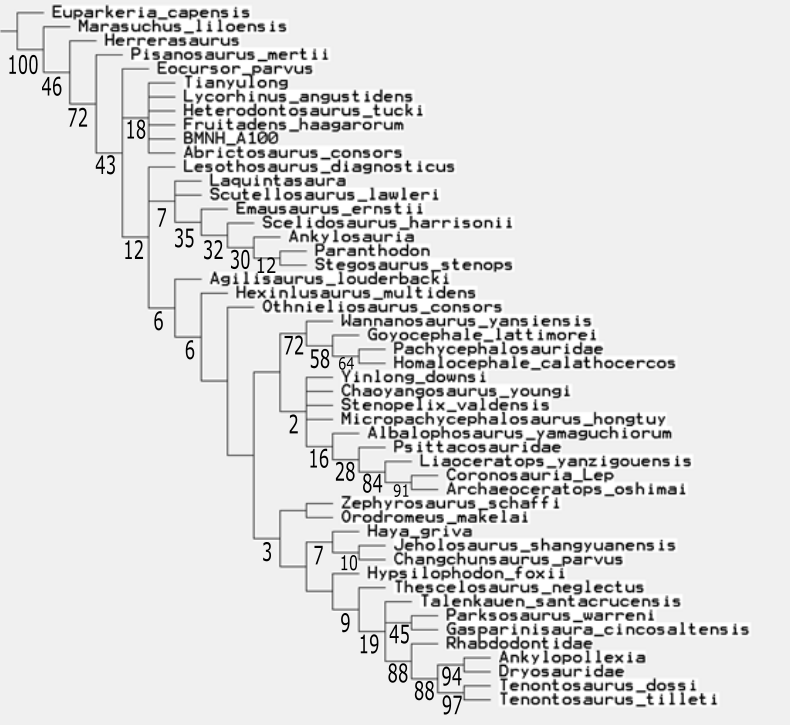


Strict consensus tree of 84 MPTs of Analysis C4


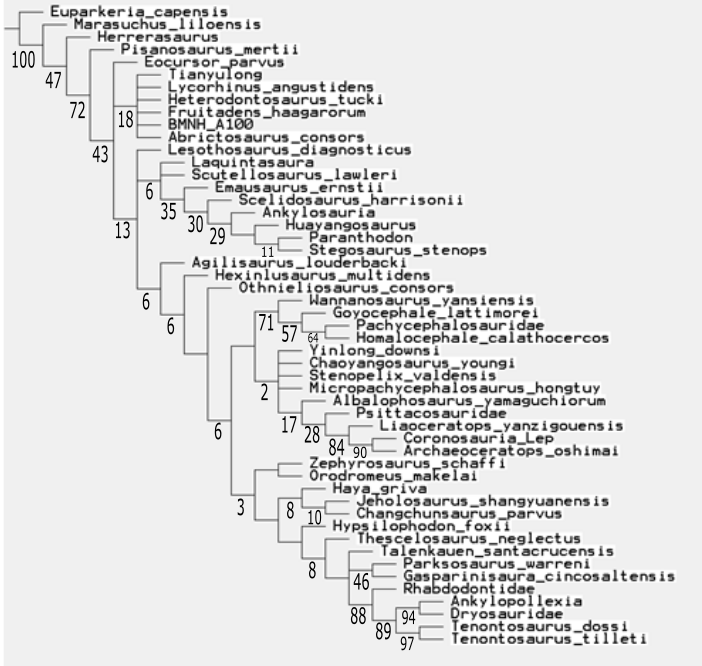


Strict consensus tree of 340 MPTs of Analysis C5


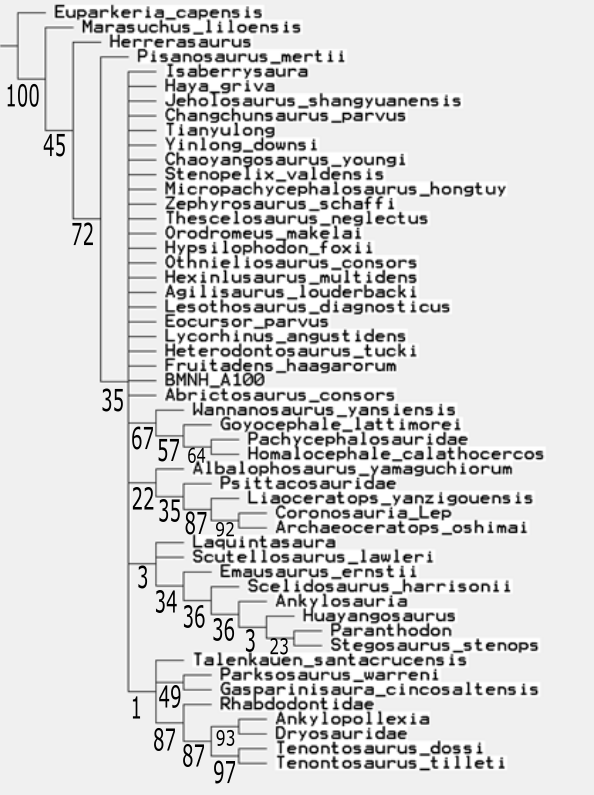


Strict consensus tree of 10 MPTs of Analysis C6


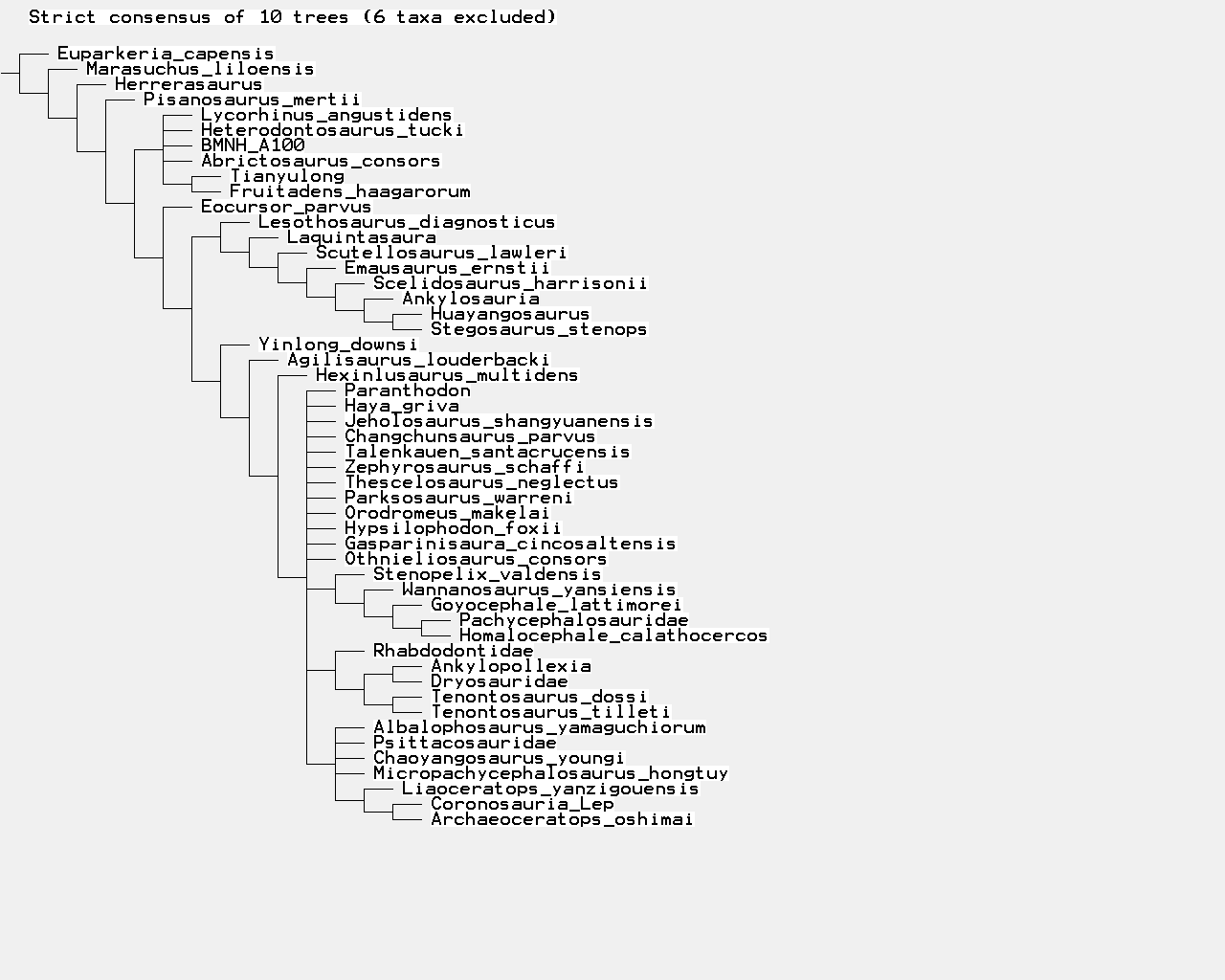


Strict consensus tree of Analysis D1


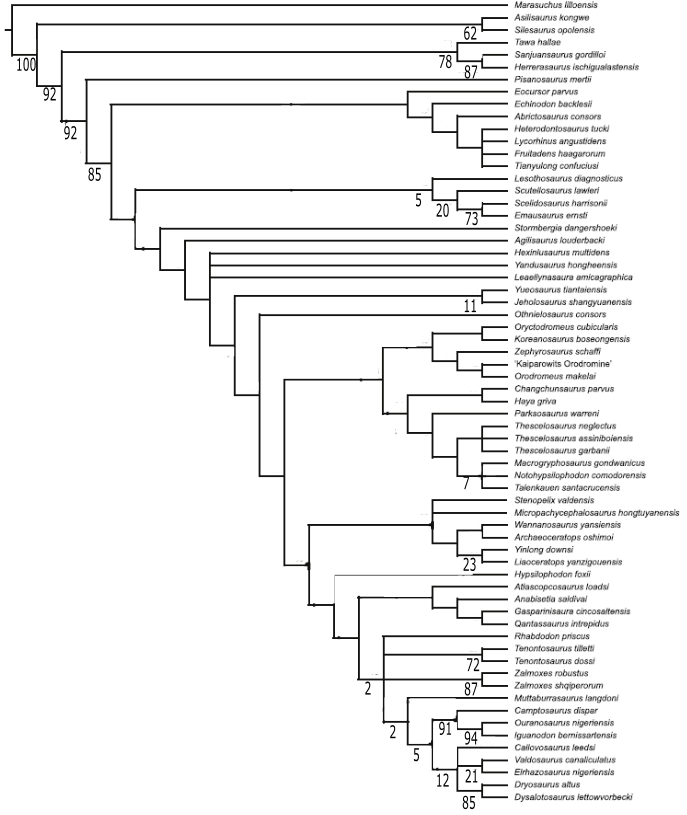


Strict consensus tree of two MPTs of Analysis D2


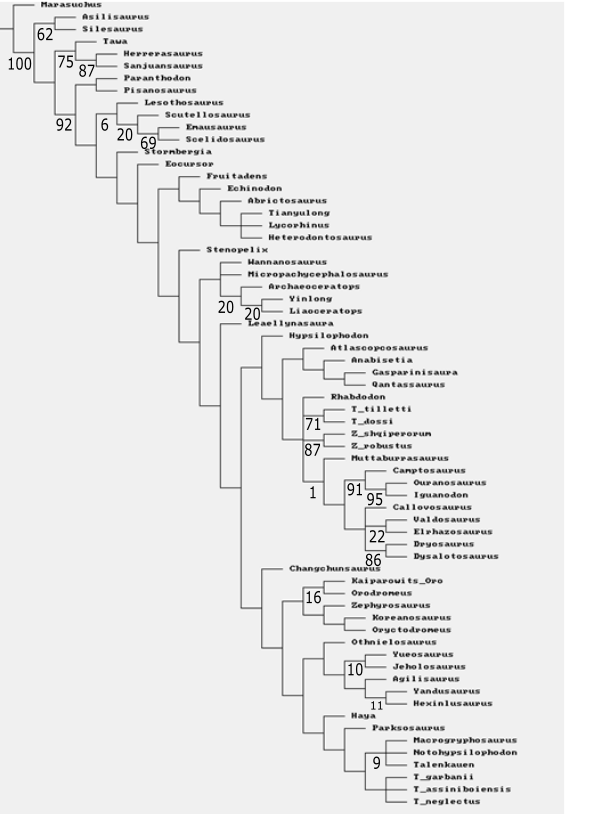


Strict consensus tree of five MPTs of Analysis D3


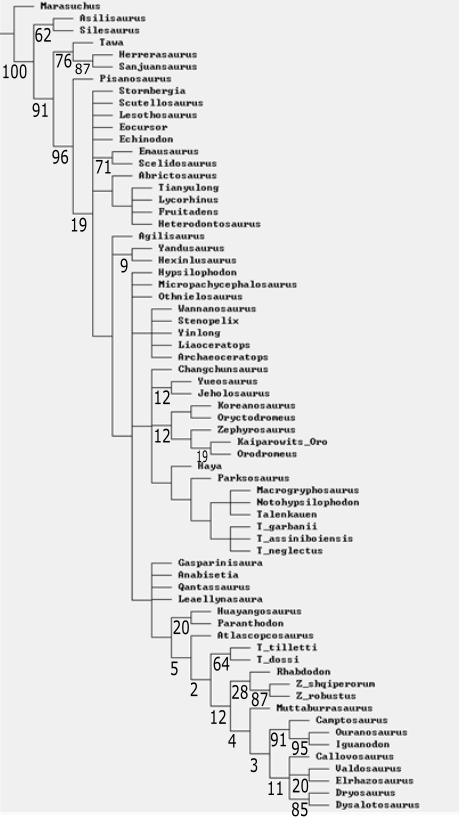


Strict consensus tree of three MPTs of Analysis D4


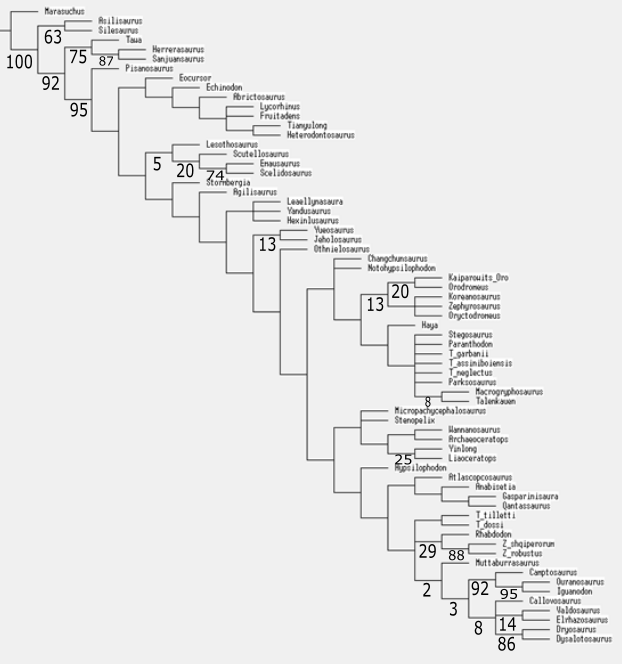


Strict consensus tree of seven MPTs of Analysis D5


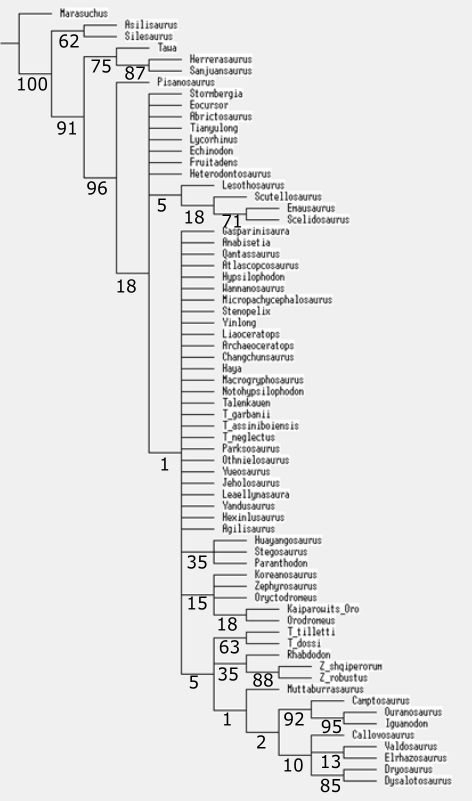


Strict consensus tree of five MPTs of Analysis D6


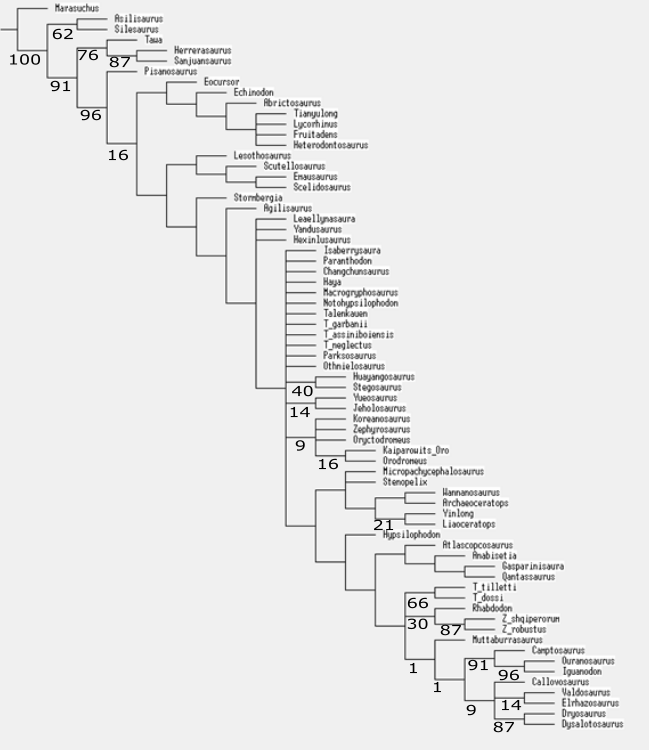


Strict consensus tree of six MPTs of Analysis D7


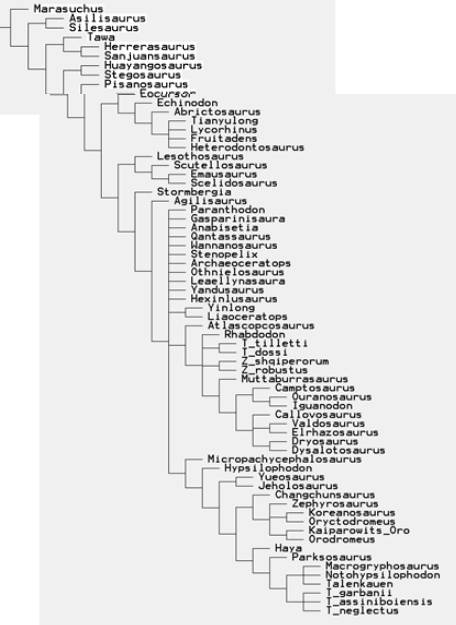


Strict consensus tree of four MPTs of Analysis D8


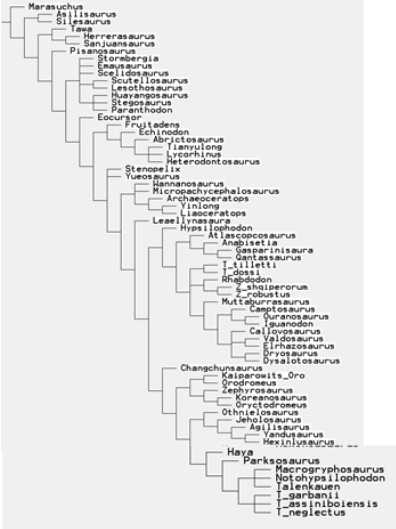


One MPT of Analysis E1


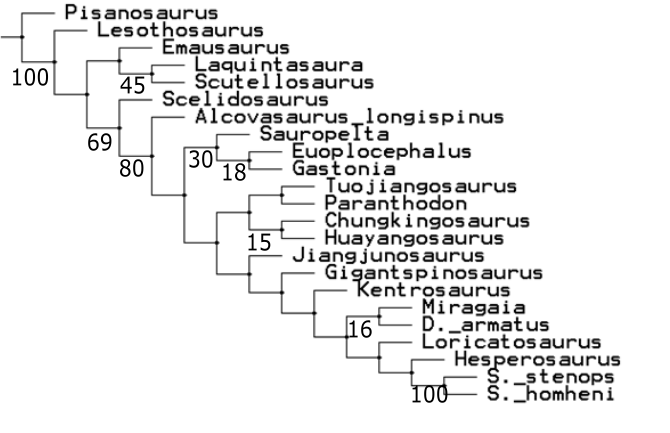


Strict consensus tree of four MPTs of Analysis E2


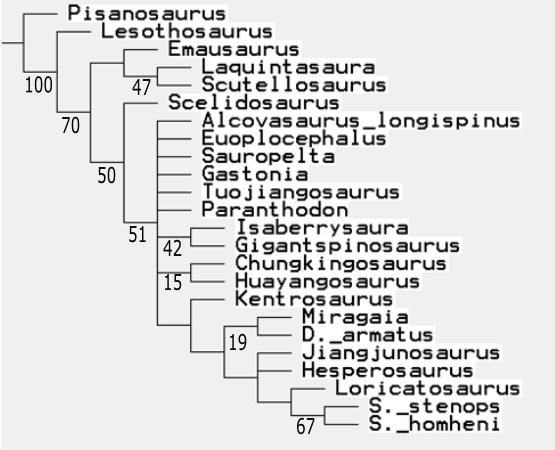


One MPT of Analysis E3


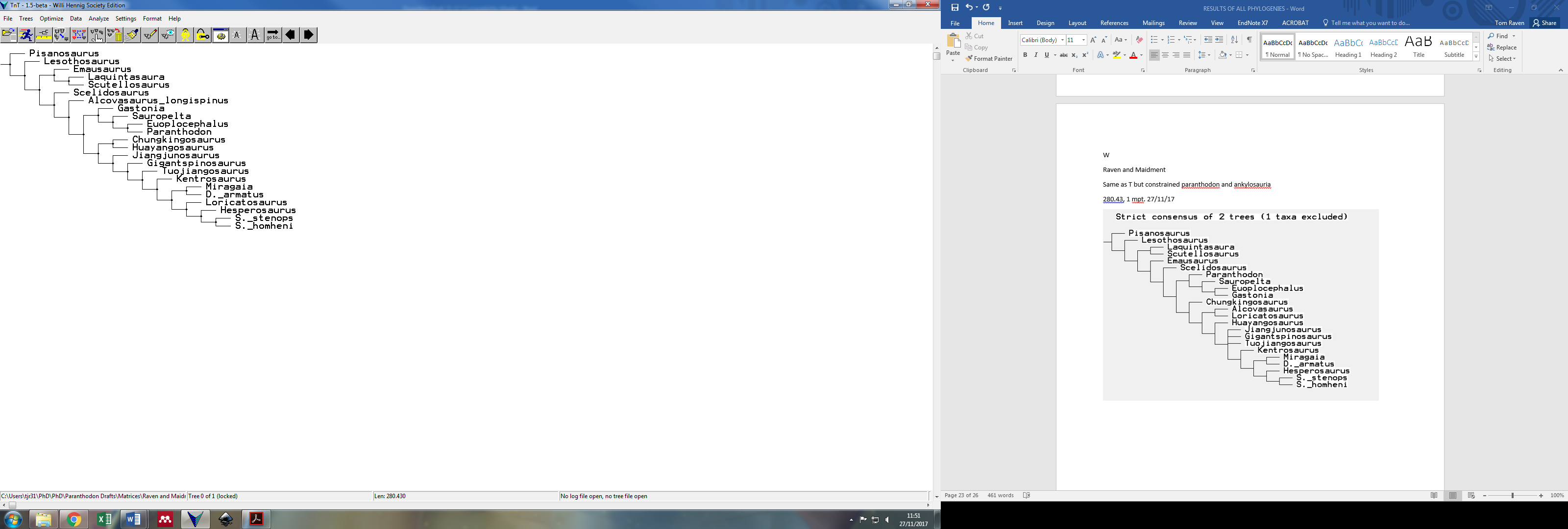


Strict consensus tree of Analysis F1


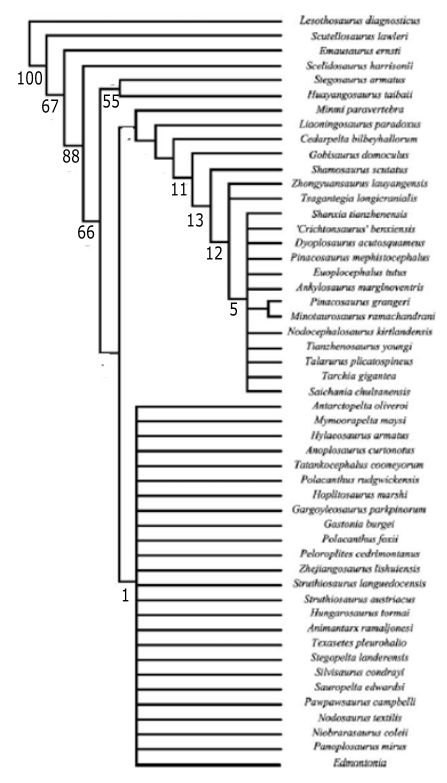


Strict consensus tree of five MPTs of Analysis F2


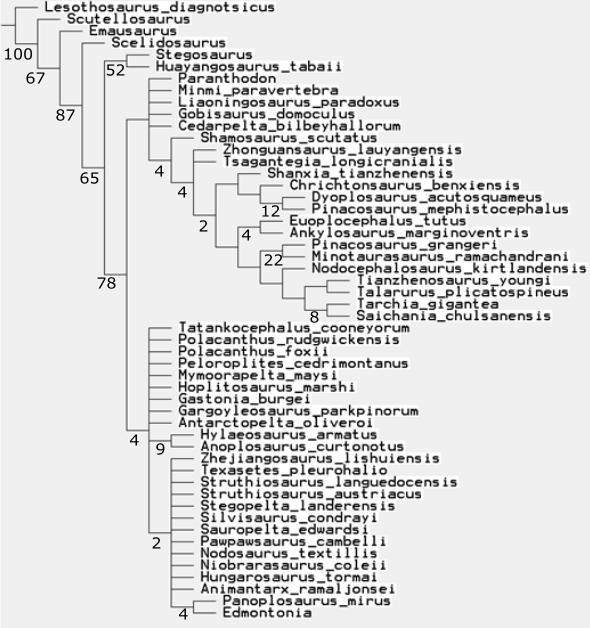


Strict consensus tree of three MPTs of Analysis F3


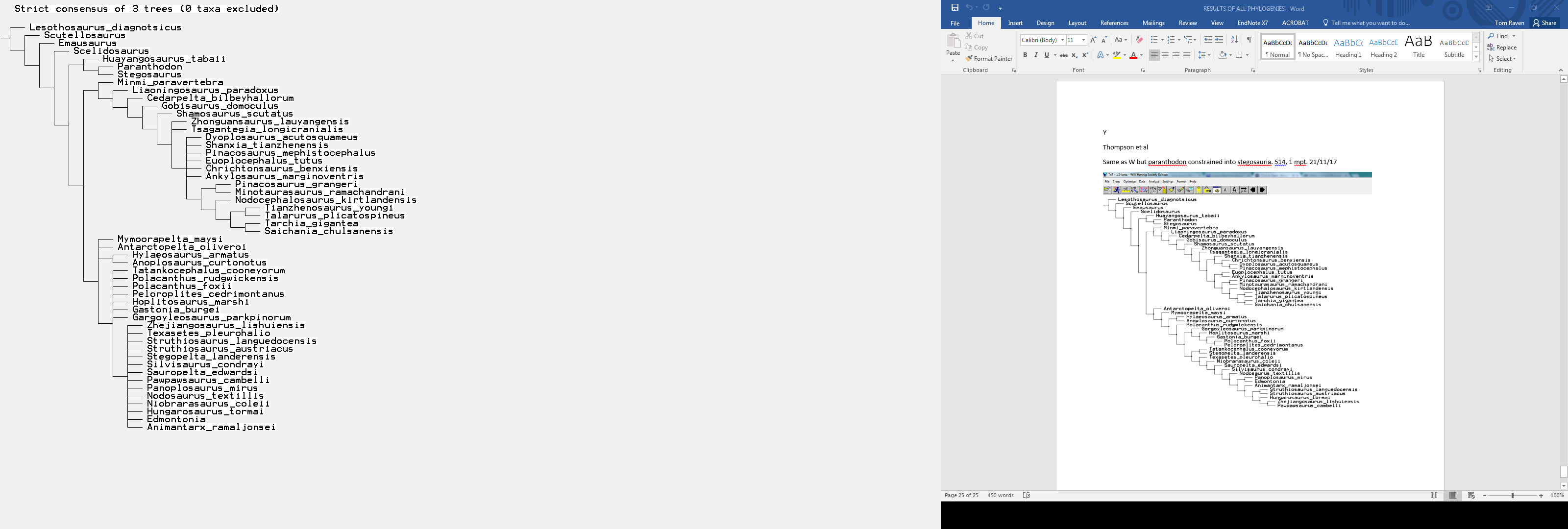


Character list for the updated Raven and Maidment (2017) analyses, with new characters highlighted

1. Snout, depth: depth to length ratio of maxilla coded as continuous.
2. Teeth: number coded as meristic.
3. Teeth: Number of denticles on mesial side of maxillary teeth.
4. Premaxilla: Height to length ratio of subnarial portion coded as continuous.
5. Cervical vertebrae: number coded as meristic.
6. Dorsal vertebrae: neural arch to neural canal height ratio as continuous.
7. Dorsal vertebrae: centrum height to neural arch height ratio coded continuously.
8. Dorsal vertebrae: centrum height to neural arch height ratio coded continuously.
9. Dorsal vertebrae: number coded as meristic.
10. Scapula: proximal plate area to coracoid area ratio coded continuously.
11. Humerus: ratio of width of distal end to minimum shaft width coded continuously.
12. Humerus: ratio of transverse width of distal end to length coded continuously.
13. Humerus: anterior iliac process length to humerus length coded continuously.
14. Ulna: proximal width to length ratio coded continuously.
15. Ratio of ulna length to humerus length coded continuously.
16. Ratio of radius length to humerus length coded continuously.
17. Metacarpal II to humerus length ratio coded continuously.
18. Ilium: anterior iliac process to acetabular length ratio coded continuously.
19. Ilium: ratio of acetabular length to dorsoventral height of pubic peduncle of ilium coded continuously.
20. Pubis: prepubis to postpubis length ratio coded continuously.
21. Pubis: postpubis to acetabular length ratio coded continuously.
22. Femur: length to humerus length ratio coded continuously.
23. Femur: length to tibia length ratio continuously.
24. Metatarsal IV: ratio of length to width coded as continuous. Maximum length and maximum widths used.
25. External nares direction. Faces anteriorly (0); faces anterolaterally (1). NEW
26. Premaxilla: Shape of subnarial portion: horizontal (0); angled ventrally (1). NEW
27. Maxilla: tooth row in ventral view sinuous (0); straight (1). NEW
28. Maxilla: medial process absent (0); present (1). NEW
29. Nasal: Curvature flat (0); gently arched (1); greatly arched (2). Code as ordered. NEW
30. Tooth crowns: striations extend to cingulum (0); do not extend to cingulum (1). NEW
31. Skull, overall shape in posterior view: deeper than wide (0); wider than deep (1).
32. Premaxilla: process projecting caudodorsally from caudolateral corner - gracile (0); robust (1).
33. Premaxilla: Caudodorsally projecting process from craniomedial border extends dorsally to be- visibile on skull roof in dorsal view (0); not visible on skull roof (1).
34. Premaxilla: Broad ‘V’ or ‘U’ shaped notch between premaxillae on the midline absent (0); present (1).
35. Maxilla: tooth row inset medially from the lateral surface of the maxilla absent (0); present (1).
36. Lacrimal: contacts prefrontal (0); doesn’t contact prefrontal (1).
37. Frontals: rostrocaudally longer than wide transversely (0); wider than long (1).
38. Frontals: form the dorsal rim of the orbit (0); supraorbital elements form the dorsal rim of the orbit (1).
39. Parietals, dorsal surface: convex (0); flat (1).
40. Quadrate: fossa/fenestra absent (0); present (1).
41. Quadrate: proximal head strongly transversely compressed, absent (0); present (1)
42. Quadrate: head is strongly arched posteriorly relative to the shaft, absent (0); present (1).
43. Quadrate: axis extending through condyles in posterior view orientated transversely (0); orientated strongly ventromedially (1).
44. Quadrate: contact with paroccipital process unfused (0); fused (1).
45. Quadrate: lateral ramus present (0); absent (1).
46. Quadratojugal: rectangular shape (0); possesses dorsal process that extends to craniolateral surface of quadrate in lateral view (1).
47. Basioccipital: exit for vagus nerve braincase: posterior surface (0); lateral surface (metotic fissure) (1).
48. Jaw joint: ventral to tooth row (0); level with tooth row (1).
49. Dentary: postdentary bones greater in rostrocaudal length than dentary (0); shorter (1).
50. Dentary: tooth row in lateral view visible (0); not visible (1).
51. Dentary: tooth alveoli face dorsally (0); dorsomedially (1).
52. Dentary: tooth row in lateral view straight (0); sinuous (1).
53. Tooth crowns: striations not confluent with denticles (0); confluent with denticles (1).
54. Tooth crowns: asymmetric (0); symmetric (1).
55. Teeth: diastema between predentary facet on the dentary and first tooth present (0); absent (1).
56. Premaxillary teeth: present (0); absent (1).
57. Maxillary teeth: cingulum absent (0); present (1).
58. Skull roof: cortical remodelling absent (0); present (1).
59. Skull roof: cortical remodelling present in only some bones (0); present in all bones, along with the fusion of dermal ossifications, so that the antorbital and supratemporal fenestrae are closed (1).
60. Axis: neural spine triangular in lateral view (0); sub-rectangular in lateral view (1).
61. Axis: ventral margin in lateral view flat (0); concave (1).
62. CV3: centrum ventral margin straight (0); concave upwards (1).
63. Cervical vertebrae: longer anterposteriorly than wide transversely (0); wider than long (1).
64. Posterior cervical vertebrae: postzygapophyses not greatly elongated (0); greatly elongated and project over the back of the posterior centrum facet (1).
65. Anterior dorsal vertebrae: prezygapophyses are separated and face each other dorsally (0); joined ventrally and face dorsomedially (1).
66. Dorsal vertebrae: cranial and caudal articular facets on centra flat to slightly concave (0); strongly convex (1).
67. Dorsal vertebrae: all centra longer than wide (0); wider than long (1).
68. Dorsal vertebrae: transverse processes project approximately horizontally (0); at a high angle to the horizontal (1).
69. Dorsasacral vertebrae ribs: don’t fuse (0); fuse to dorsal margins of first true sacral vertebrae (1); fuse to medial margin of preacetabular process of ilium (2).
70. Sacral rod vertebrae: keel present (0); absent (1).
71. Anterior caudal vertebrae: dorsal process on transverse process absent (0); present (1).
72. Anterior caudal vertebrae: dorsal process on transverse process proximal to centrum (0); distal to centrum (2).
73. Anterior caudal vertebrae: transverse processes on cd3 posteriorly are directed laterally (0); directed strongly ventrally (1).
74. Anterior caudal vertebrae: neural spine height less than or equal to the height of the centrum (0); greater than the height of the centrum (1).
75. Anterior caudal vertebrae: bulbous swelling at tops of neural spines absent (0); present (1).
76. Caudal vertebrae: prezygapophyses extend craniodorsally (0); extend cranially (1).
77. Caudal vertebrae: postzygapophyses extend cranially over caudal articular facet (0); do not (1).
78. Caudal vertebrae: transverse processes on distal half of tail present (0); absent (1).
79. Caudal vertebrae: neural spines bifurcated (0); not bifurcated (1).
80. Posterior caudal vertebrae: centra are elongate (0); equidimensional (1).
81. Scapula: acromial process in lateral view, convex upwards dorsally (0); quadrilateral with a posterordorsal corner (1).
82. Scapula: acromial process projects dorsally (0); projects laterally (1).
83. Scapula: blade, distally expanded (0); parallel sided (1).
84. Coracoid: sub-circular outline (0); anteroposteriorly longer than dorsoventrally high (1).
85. Coracoid: in lateral view, foramen present (0); notch present (1).
86. Humerus: triceps tubercle and descending ridge posterolateral to the deltopectoral crest absent (0); present (1).
87. Radius: expanded transversely at proximal end (0); not expanded (1).
88. Metacarpals I and V: shorter than metacarpals II, III and IV (0); longer (1).
89. Ungual phalanges: Manual and pedal unguals claw–shaped (0); hoof–shaped (1).
90. Ilium: anterior iliac process lies approximately horizontally (0); strongly angled ventrally (1).
91. Ilium: anterior iliac process projects roughly parallel to the parasagittal plane (0); diverges widely from the parasagittal plane (1).
92. Ilium: horizontal lateral enlargement absent (0); present (1).
93. Ilium: horizontal lateral enlargement incipient (small) (0); large (1).
94. Ilium: supra–acetabular flange projects at 90 degrees from the anterior iliac process absent (0); present (1).
95. Ilium: posterior iliac process, distal shape tapers (0); blunt (1).
96. Ilium: medial processes on posterior iliac processes absent (0); present (1).
97. Ilium: ventromedial flange backing the acetabulum absent (0); present (1).
98. Ilium: preacetabular process has inverted C-shaped cross section that is laterally convex and medially concave (0); does not i.e. transversely compressed (1).
99. Ilio–sacral block: Five or more sacral vertebrae (0); four or fewer sacral vertebrae (1).
100. Ilio–sacral block: Posterior sacral rib angled laterally (0); posterolaterally (1).
101. Ilio–sacral block: dorsal shield of sacrum is perforated by foramina in between ribs (0); is solid with no foramina (1).
102. Ischium: convex proximal margin within the acetabulum absent (0); present (1).
103. Ischium: dorsal surface of shaft is straight (0); has a distinct angle at approximately midlength (1).
104. Ischium: posterior end of ischium, expanded relative to the shaft (0); not expanded and tapers (1).
105. Pubis: obturator notch is backed by posterior pubic process absent (0); present (1).
106. Pubis: acetabular portion faces laterally, posteriorly and dorsally (0); faces wholly laterally (1).
107. Pubis: anterior end of prepubis expanded dorsally absent (0); present (1).
108. Femur: Fourth trochanter prominent and pendant (0); present as a rugose ridge (1); absent (2).
109. Femur: anterior trochanter fusion to greater trochanter in adults - unfused (0); fused (1).
110. Metatarsal V: present (0); absent (1).
111. Pedal digit I: present (0); absent (1).
112. Pedal digit III: has 4 or more phalanges (0); has 3 phalanges (1); has 2 or fewer phalanges (2). Code as ordered.
113. Pedal digit IV: has 5 phalanges (0); has 4 phalanges (1); has 3 or fewer phalanges (2). Code as ordered.
114. Dermal armour: including scutes, and/or spines and/or plates absent (0); present (1).
115. Plates and spines: two parasagittal rows of plates and/or spines absent (0); present (1).
116. Cervical collars: U–shaped cervical collars composed of keeled scutes absent (0); present (1).
117. Osteoderms: mosaic of small osteoderms between larger osteoderms on the ventral surfaces of the neck, trunk, and proximal portions of the limbs absent (0); present (1).
118. Parascapular spine: absent (0); present (1).
119. Dorsal plates: have a thick central portion like a modified spine (0); have a generally transversely thin structure, except at the base (1).
120. Parasagittal rows of dermal armour: paired (0); alternating either side of the midline (1).
121. Ossified epaxial tendons: present (0); absent (1).

Characters from Boyd (2015) that unite either Eurypoda, Eurypoda + *Alcovasaurus* or Stegosauria in Raven and Maidment (2017)

- Character 23: Supraorbital absent (0), one supraorbital present (1), two or more supraorbitals present (2). This is similar to Character 32 of Raven and Maidment (2017).
- Character 52: Distal condyles of the quadrate dorsomedially sloped or horizontally oriented (0), distal condyles of the quadrate dorsolaterally sloped (1). This is similar to Character 37 of Raven and Maidment (2017).
- Character 111: Premaxillary teeth present (0), absent (1). This is the same as Character 50 of Raven and Maidment (2017).
- Character 129: Cingulum on maxillary teeth present (0), cingulum on maxillary teeth absent (1). This is the same as Character 51 of Raven and Maidment (2017).
- Character 161: Coracoid foramen enclosed within the coracoids (0), coracoid foramen open along coracoid-scapula articular contact surface (1). This is similar to Character 79 of Raven and Maidment (2017).
- Character 219: Fourth trochanter of the femur ‘mound-like’ (0), fourth trochanter a sharp ridge (1), fourth trochanter ‘pendant-shaped’ (2), fourth trochanter subtriangular (3), fourth trochanter vestigial, consisting of a rugosity or scar (4). This is similar to Character 102 of Raven and Maidment (2017).
- Character 244: Metatarsal V present (0), absent (1). This is the same as Character 104 of Raven and Maidment (2017).
